# Effect of Flow Velocity on Laminar Flow in Microfluidic Chips

**DOI:** 10.3390/mi14071277

**Published:** 2023-06-21

**Authors:** Chuang Wu, Haithm Yahya Mohammed Almuaalemi, A. S. M. Muhtasim Fuad Sohan, Binfeng Yin

**Affiliations:** 1 School of Mechanical Engineering, Yangzhou University, Yangzhou 225127, China; mh22012@stu.yzu.edu.cn; 2 Nantong Fuleda Vehicle Accessory Component Co., Ltd., Nantong 226300, China; 3 Jiangsu Tongshun Power Technology Co., Ltd., Nantong 226300, China; 4 Faculty of Engineering, Department of Mechanical Engineering, University of Adelaide, Adelaide, SA 5000, Australia; asmmuhtasimfuad.sohan@student.adelaide.edu.au

**Keywords:** microfluidic technology, laminar flow, gel fiber, fluid dynamics, sodium alginate

## Abstract

Gel fibers prepared based on microfluidic laminar flow technology have important research value in constructing biomimetic scaffolds and tissue engineering. The key point of microfluidic laminar flow technology is to find the appropriate fluid flow rate in the micropipe. In order to explore the influence of flow rate on the laminar flow phenomenon of a microfluidic chip, a microfluidic chip composed of an intermediate main pipe and three surrounding outer pipes are designed, and the chip is prepared by photolithography and the composite molding method. Then, a syringe pump is used to inject different fluids into the microtubing, and the data of fluid motion are obtained through fluid dynamics simulation and finite element analysis. Finally, a series of optimal adjustments are made for different fluid composition and flow rate combinations to achieve the fluid’s stable laminar flow state. It was determined that when the concentration of sodium alginate in the outer phase was 1 wt% and the concentration of CaCl_2_ in the inner phase was 0.1 wt%, the gel fiber prepared was in good shape, the flow rate was the most stable, and laminar flow was the most obvious when the flow rate of both was 1 mL/h. This study represents a preliminary achievement in exploring the laminar flow rate and fabricating gel fibers, thus offering significant reference value for investigating microfluidic laminar flow technology.

## 1. Introduction

Micro-nano-sized gel fibers with a large specific surface area hold great promise for applications in the biomedical field, enabling the simulation of in vivo microenvironments [1,2,3]. Currently, various methods are employed to prepare gel fibers, including electro-spinning technology [4,5,6], wet-spinning technology [7,8,9], and microfluidic technology [10,11,12,13,14]. Nagrath et al. [15] successfully developed bioactive gel fibers by combining the sol-gel method with electro-spinning technology. These fibers serve as carriers for bone regeneration, cell proliferation, wound healing, and drug delivery. Due to their biomimetic properties, they have broad potential in soft tissue repair and angiogenesis. Similarly, Zheng et al. [16] utilized electro-spinning technology to create an artificial neural catheter using PLLA and porcine decellularized neural matrices as materials. Through histological evaluation and immunostaining analysis in rat implants, they demonstrated that the artificial neural catheter promoted nerve axon extension and nerve fiber formation. Furthermore, Xu et al. [17] employed wet electro-spinning technology to produce nano-reinforced ceramic composite fibers in situ. These fibers retained the amorphous structure of the material, increased porosity, and improved fracture toughness, resulting in significant hardening and toughening effects on polymer composites. However, both electro-spinning and wet spinning methods can potentially damage cells when perfused during fiber preparation [18,19,20].

Microfluidic laminar flow technology has emerged as a novel approach for gel fiber fabrication [21,22]. Nguyen et al. [23] utilized polydimethylsiloxane (PDMS) microfluidic chips to produce tadpole-shaped alginate gel fibers, in which mesenchymal stem cells (MSCs) were implanted. By inducing chondrocyte differentiation using transforming growth factors, they achieved a chondrocyte composition of 64% after 21 days of differentiation. Moreover, the high viability of cells was observed after 95 days of culture. Some researchers [24,25,26] developed a microfluidic device based on glass capillaries, employing an inner layer of photopolymerizable material and an outer layer of non-photo polymeric material. This design ensured that the fluids remained separated until ultraviolet light irradiation at the outlet, solidifying the material into gel fibers. Additionally, other studies [27,28,29] proposed using sodium alginate as the inner layer material and calcium chloride (CaCl_2_) as the outer layer material for ion polymerization-based gel fiber preparation.

However, microfluidic technology necessitates highly controlled laminar flow of fluids within the microchannels, particularly for continuous production of hierarchical gel fibers [30,31,32]. Hence, it is crucial to explore different flow rate combinations to achieve stable laminar flow phenomena. In this study, we present a microfluidic chip device with a simple structure and convenient operation, facilitating observation of fluid laminar flow states within the chip. Finite element analysis is employed to determine the composition of fluid flow velocities in the laminar flow state. This analysis contributes to the study of gel fiber preparation using microfluidic laminar flow technology.

## 2. Materials and Methods

### 2.1. Materials

The PDMS (Sylgard 184) was obtained from Dow Corning, headquartered in Midland, MI, USA. Ultra-pure water, polyvinyl alcohol (PVA), rhodamine B, sodium alginate, CaCl_2_, and anhydrous ethanol were procured from Aladdin Biochemical Technology, situated in Shanghai, China. The photoresist (AZ-50XT) and developer were sourced from AZ Electronic Materials, situated in Somerville, NJ, USA.

### 2.2. Methods

#### 2.2.1. Preparation of Microfluidic Chips

**Figure 1:**
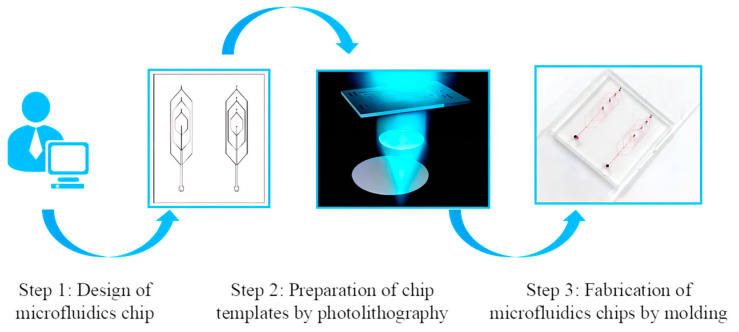
The preparation process of a microfluidic chip.

Step 1: The microfluidic chip was designed using Solidworks® version 2021 software (Dassault Systèmes SolidWorks Corporation, Coventry, UK), as depicted in Figure 1. The chip consists primarily of two types of channel. The central vertical channel serves the purpose of introducing the inner phase fluid, which contributes to the formation of a hollow fiber structure. The main pipe size gradually increases from top to bottom, starting from 50 μm and progressing to 100, 150, and finally 200 μm, following a narrowing-to-widening pattern. The outer region of the chip features a three-ring annular channel, specifically designed for introducing the external phase fluid responsible for forming the fibers. The diameter of the circular channel is set to 50 μm.

Step 2: The manufacturing process of the microfluidic chip template involved the fabrication of the micro-structure using photolithography and a photoresist (AZ-50XT). Initially, the silicon wafer was immersed in a prepared Piranha solution for a duration of 15 min. Subsequently, the silicon wafer was rinsed successively with ultrapure water, absolute ethanol, acetone, and ultrapure water. Following this cleaning process, the silicon wafers were subjected to a 5-min baking step to eliminate any residual moisture from the surface. Additionally, a 5-min fumigation with trimethylchlorosilane (TMSC) was performed. Finally, a series of sequential operations were carried out, including silicon wafer coating, silicon wafer bonding, silicon wafer baking, silicon wafer exposure, and silicon wafer development, to create the desired microstructure for the microfluidic chip template.

Step 3: In order to prepare the microfluidic chip, the fabrication process involved the utilization of Polydimethylsiloxane (PDMS) materials through photolithography. Initially, a PDMS solution was prepared by combining PDMS with a curing agent in a ratio of 10:1. Subsequently, a TMSC fumigated silicon wafer template was subjected to a 3-min injection pumping process, followed by curing, peeling, cutting, and punching. Ultimately, the PDMS microfluidic chip was packaged by adjusting the temperature to 80 °C and allowing it to bake for a duration of 72 h.

#### 2.2.2. Fluid Injection Mode

**Figure 2:**
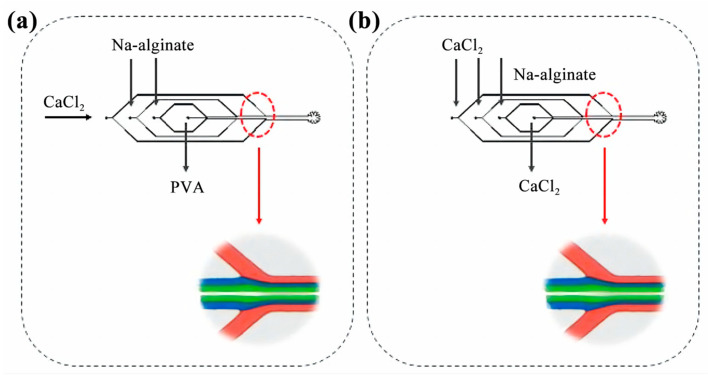
Fluid injection: (**a**) the first injection method; (**b**) the second injection method.

In order to achieve laminar flow, it is necessary to introduce each fluid into the system through distinct microtube inlets, while maintaining a constant flow rate. Once injected, the fluid gradually travels through the micro duct until it reaches the intersection point. There are two distinct methods of injection, as depicted in Figure 2:

In the first approach (Figure 2a), the intermediate pipeline contains a 10 wt% PVA solution, while the inner two layers consist of a 1 wt% sodium alginate solution. The outermost layer is composed of a 0.1 wt% CaCL_2_ solution.

In the second approach (Figure 2b), the middle pipe and the outer two layers are filled with a 0.1 wt% CaCL_2_ solution, while the innermost layer comprises a 1 wt% sodium alginate solution.

#### 2.2.3. Numerical Simulation of Fluid Motion

To explore the influence of flow rate on the laminar flow phenomenon in microfluidic chips, we utilized ANSYS (version 2021 R1) for performing finite element analysis of the fluid dynamics within the microfluidic chip. A finite element model was constructed, considering the pipe wall as a non-sliding boundary condition. This model allowed us to investigate the changes in flow velocity occurring within the micropipe.

#### 2.2.4. Effect of Flow Rate on Laminar Flow Phenomenon

In the experiment, the fluid’s flow rate was divided into two components: the flow rate of the middle main pipe and the flow rate of the outer three layers of the pipeline. In the first experiment, five different combinations of flow velocities were established, where the flow rate of the outer layer was twice that of the intermediate flow rate (Table 1). The second experiment involved setting up ten different combinations of flow velocities, with the internal and external flow velocities being the same (Table 2). The third experiment comprised four different combinations of flow velocities. In this case, the flow rate of the main intermediate pipeline remained unchanged, while the flow rate of the outer layer was gradually increased (Table 3). To observe the laminar flow state, the fluid in the experiment was stained with pigment.

#### 2.2.5. Characterization of Laminar Flow Phenomena

Characterize fluids within microfluidic chip tubing under conditions of stable fluid motion.

The perfused fluid is dyed separately with pigments and, from the inside to the outside, is colorless, green, blue, and red. Using a volume microscope, a brightfield intersection map was taken to observe the laminar flow state. The perfused fluid was stained with rhodamine B fluorescent dye, and the fluorescence map of the intersection was taken using an inverted fluorescence microscope (Eclipse Ti-U, Nikon, Japan) to observe the laminar flow state.

#### 2.2.6. Statistical Analysis

All data are presented as mean ± one standard deviation (SD) of n samples for each experimental group. Groups were compared using one-way analysis of variance (ANOVA) to determine significance. Differences between groups were considered significant when *p* < 0.05.

## 3. Results and Discussion

### 3.1. Microfluidic Chip

**Figure 3:**
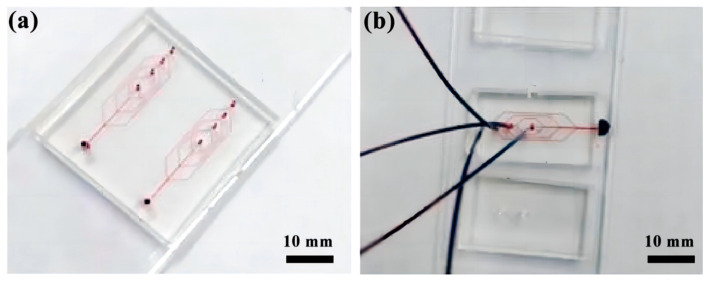
Microfluidic chip fluid experiment diagram: (**a**) Microfluidic chip; (**b**) Stained fluid injection.

Using a 1 mL syringe, the stained fluid is injected through a syringe pump into the microtubing of the microfluidic chip to explore the state of fluid flow (Figure 3).

### 3.2. Finite element Model of Fluid Flow Velocity

**Figure 4:**
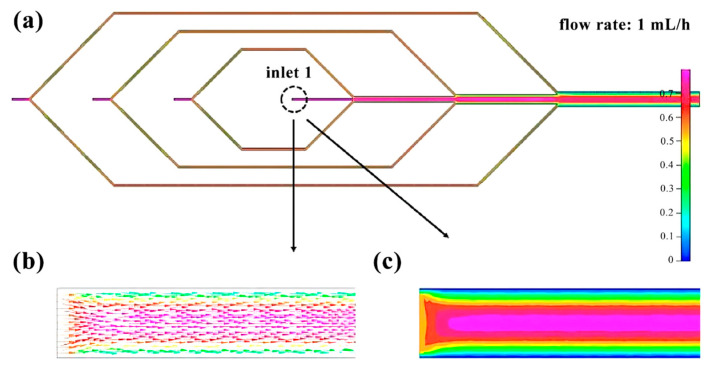
Finite element diagram of microfluidic chip inlet 1: (**a**) Full picture; (**b**) Flow direction simulation diagram of inlet 1; (**c**) Flow velocity distribution diagram of inlet 1.

**Figure 5:**
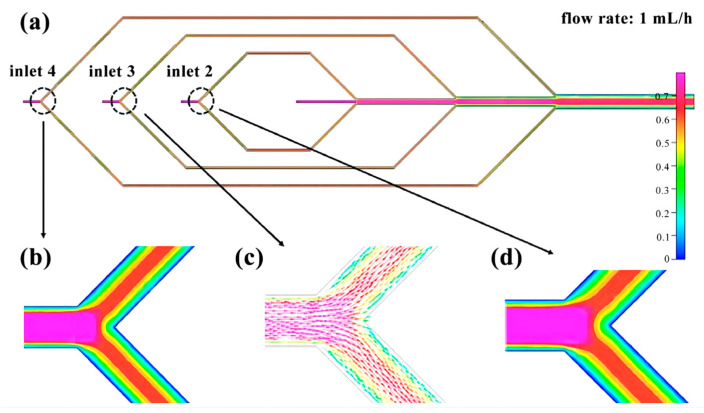
Finite element diagram of microfluidic chip from inlet 2 to inlet 4: (**a**) Full picture; (**b**) Velocity distribution diagram of inlet 4; (**c**) Flow direction simulation diagram of inlet 3; (**d**) Velocity distribution diagram of inlet 2.

**Figure 6:**
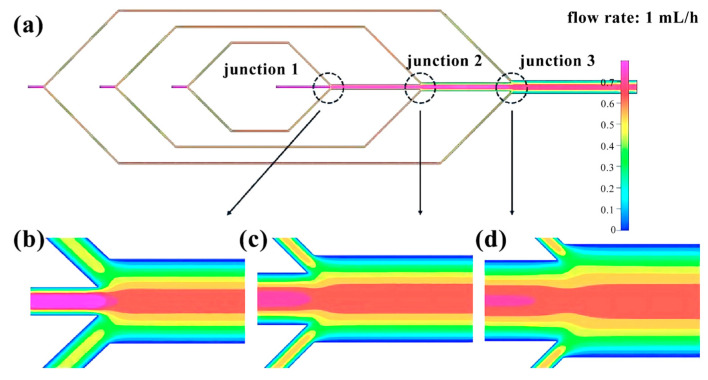
Finite element diagram of microfluidic chip intersection: (**a**) Full picture; (**b**) Flow velocity distribution at junction 1; (**c**) Flow velocity distribution at junction 2; (**d**) Flow velocity distribution at junction 3.

The flow rate of the four inlets of the microfluidic chip is set at 1 mL/h. By utilizing finite element analysis, we can determine the change in flow rate within the microchannel. Inlet 1 (Figure 4) exhibits relatively stable fluid movement. Inlets 2 to 4 (Figure 5) experience a division of flow rate upon entry, with the fluid velocity remaining steady within the pipeline. However, following the division, the flow velocity gradually decreases from the center of the pipe to the pipe wall. The flow rate remains relatively stable before and after the three intersections, but gradually decreases from the center of the pipe to the pipe wall (Figure 6).

**Figure 7:**
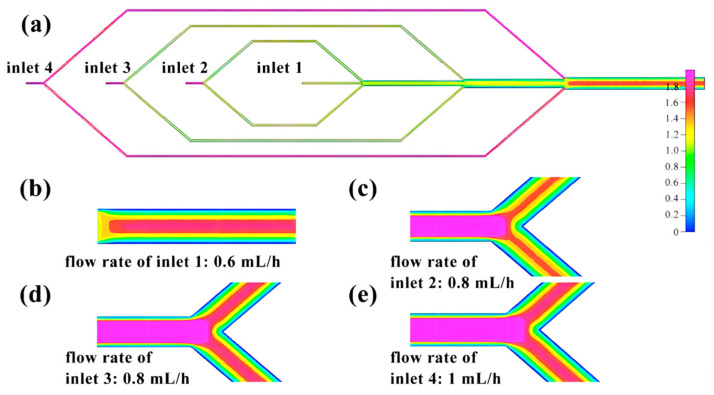
Finite element diagram of microfluidic chip inlet: (**a**) Full picture; (**b**) Flow velocity distribution at inlet 1; (**c**) Flow velocity distribution at inlet 2; (**d**) Flow velocity distribution diagram of inlet 3; (**e**) Flow velocity distribution diagram of inlet 4.

**Figure 8:**
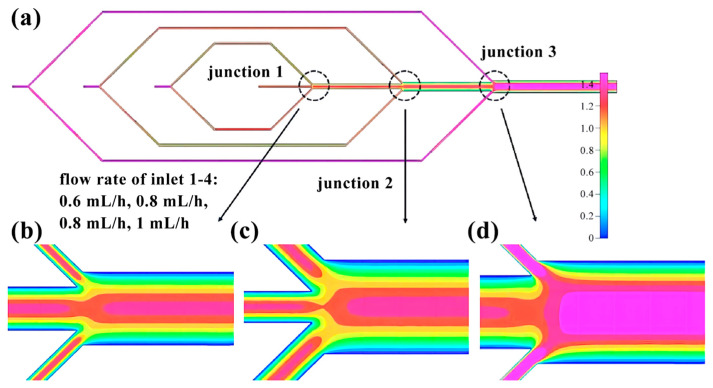
Finite element diagram of microfluidic chip intersection: (**a**) Full picture; (**b**) Flow velocity distribution at intersection 1; (**c**) Flow velocity distribution at intersection 2; (**d**) Flow velocity distribution at intersection 3.

The flow rates of the four inlets of the microfluidic chip, from the inside to the outside, are 0.6 mL/h, 0.8 mL/h, 0.8 mL/h, and 1 mL/h, respectively. Through finite element analysis, it is determined that regardless of the presence or absence of a shunt, the distribution of flow velocity exhibits a pattern of higher velocities at the center of the pipe, gradually decreasing towards the wall (Figure 7). The flow rate remains relatively stable before and after the three intersections, but gradually decreases from the center of the pipe to the pipe wall upon entering the main pipeline (Figure 8).

### 3.3. Effect of Flow Rate on Laminar Flow Phenomenon

**Figure 9:**
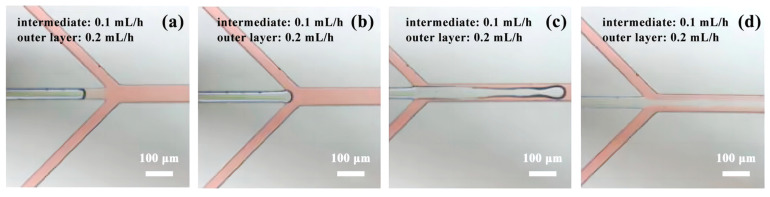
Experiment 1: Group 1: phenomenon at the intersection of fluids: (**a**–**c**) Intersection process; (**d**) Laminar flow phenomenon.

**Figure 10:**
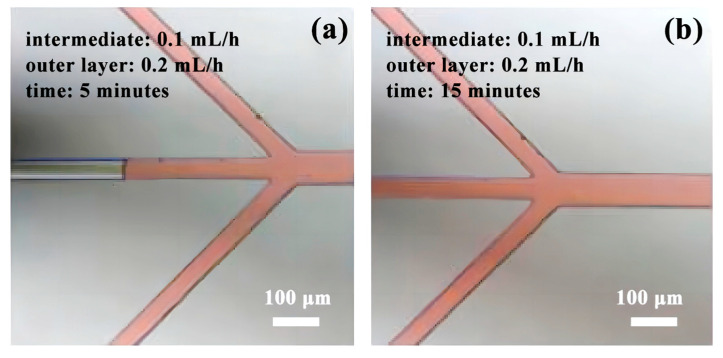
Experiment 1: Fluid backflow phenomenon in group 1: (**a**) Outer fluid backflow phenomenon; (**b**) Outer fluid completely backflows into the middle pipeline.

In Experiment 1, consisting of five datasets, the initial group exhibited a laminar flow pattern upon reaching the intersection point (see Figure 9). However, after a continuous flow period of 5 min, the microfluidic chip experienced fluid backfilling (as depicted in Figure 10a). Further observation for an additional 15 min revealed a diminishing presence of laminar flow and an intensified occurrence of fluid backfilling (as depicted in Figure 10b). This pattern persisted in the subsequent four experimental scenarios involving different flow rate combinations. Although all groups initially displayed laminar flow, they were unable to sustain this state over an extended duration. The possible reason is that the flow rate of the outer layer is higher than that of the inner layer, and there is a partial vacuum inside the pipeline. At the same time, due to the capillary action, the fluid flows backward at a low speed.

**Figure 11:**
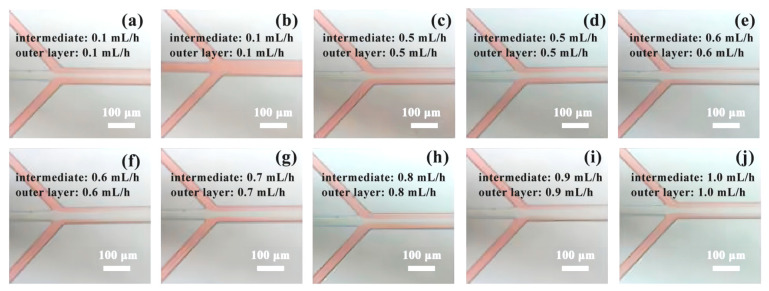
The fluid phenomenon in Experiment 2: (**a**,**b**) Backfilling of the outer fluid when the flow rate is 0.1 mL/h; (**c**,**d**) Stable laminar flow state when the flow rate is 0.5 mL/h; (**e**,**f**) Stable laminar flow state when the flow rate is 0.6 mL/h; (**g**) Stable laminar flow state when the flow rate is 0.7 mL/h; (**h**) Stable laminar flow state when the flow rate is 0.8 mL/h; (**i**) Stable laminar flow state when the flow rate is 0.9 mL/h; (**j**) Stable laminar flow state when the flow rate is 1.0 mL/h.

Within Experiment 2, comprising ten datasets, Group 1 encountered fluid backfilling in the pipeline after 15 min (illustrated in Figure 11a,b). This phenomenon also manifested in groups 2 to 4. However, in Group 5, laminar flow was observed (depicted in Figure 11c) and maintained stability beyond the 15-min mark (as shown in Figure 11d). Subsequent groups (Figure 11e–j) exhibited consistent and stable laminar flow states. The possible reason is that at low speed, the forward force of the fluid is less than the adsorption force of the liquid surface on the solid, resulting in capillary action, which causes the flowback.

**Figure 12:**
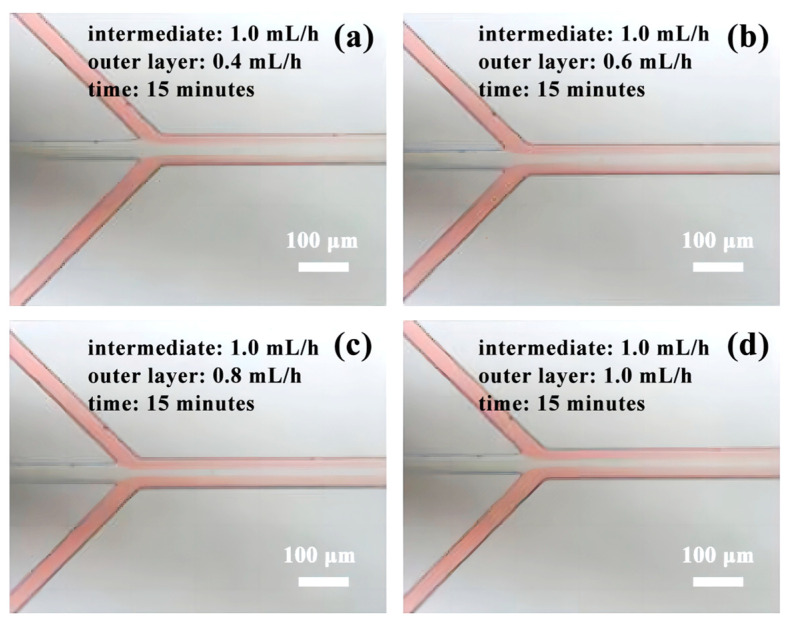
The fluid phenomenon in experiment 3: (**a**) laminar flow state of group 1; (**b**) laminar flow state of group 2; (**c**) laminar flow state of group 3; (**d**) laminar flow state of group 4.

Among the four datasets obtained from Experiment 3, the first group exhibited stable laminar flow for a duration of 15 min (as indicated in Figure 12a). Groups 2 to 4 displayed relatively stable laminar flow states, aside from slight variations in the width of the intermediate unstained fluid (depicted in Figure 12b–d).

Comparatively, when examining the three aforementioned experiment types, it becomes evident that the fluid movement is most stable and the laminar flow phenomenon is more pronounced when the internal and external flow velocities are set at 1.0 mL/h. These findings align more closely with the data derived from the fluid simulation.

### 3.4. Characterization of the Fluid Laminar Flow Phenomenon

**Figure 13:**
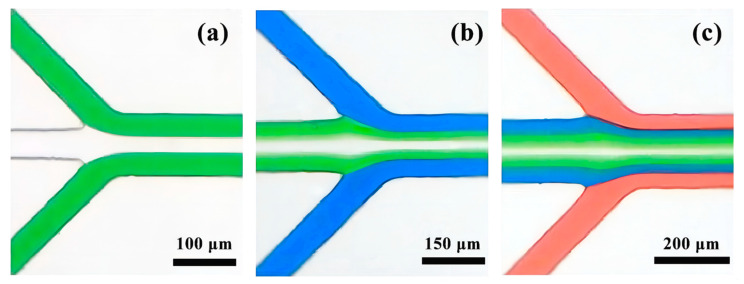
Brightfield diagram of the stained fluid intersection: (**a**) Laminar flow phenomenon at junction one; (**b**) Laminar flow phenomenon at junction 2; (**c**) Laminar flow phenomenon at junction 3.

The stability of fluid movement is maximized when the internal and external flow rates are maintained at 1.0 mL/h. Upon their convergence at the initial junction, it can be observed that the fluid seamlessly enters the central pipe without undergoing fusion, as depicted in Figure 13a. As the three fluids continue to progress, they remain in a stable laminar flow state, entering the central pipe through the second junction without any disturbances, as illustrated in Figure 13b. Upon reaching the preceding junction, the four fluids traverse smoothly and uniformly, with distinct boundaries between each fluid, as depicted in Figure 13c.

**Figure 14:**
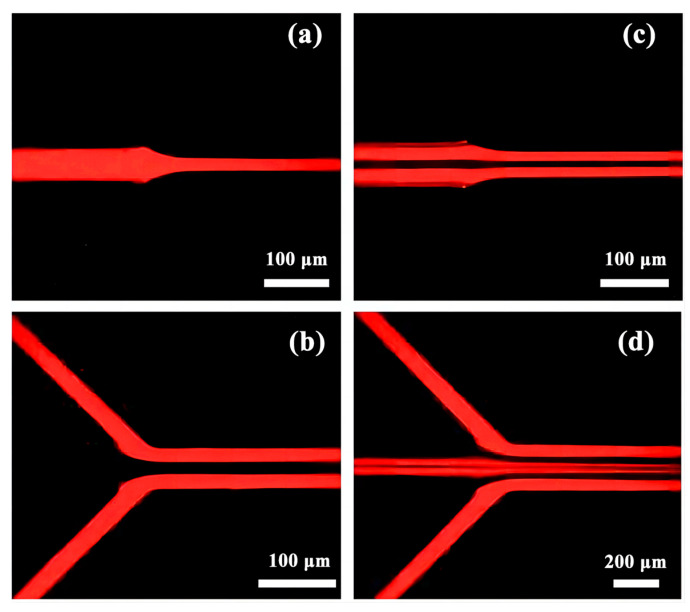
Fluorescence map of staining fluid intersections: (**a**) Fluorescence map of inner layer fluid at intersection 1; (**b**) Fluorescence map of outer layer fluid at intersection 1; (**c**) Fluorescence map of three fluid intersections at intersection 2; (**d**) Fluorescence map of four fluid intersections at intersection 3.

Within the intermediate pipeline, a fluid containing rhodamine B as a dopant is introduced. Upon their encounter at the initial junction, it is observed that the fluid effortlessly proceeds to the subsequent section of the central pipeline without merging with the other fluid, as shown in Figure 14a. The outer layer of pipes is filled with a rhodamine B-doped fluid, resulting in a stable laminar flow state at the first junction, as depicted in Figure 14b. This laminar flow state is maintained as the three fluids converge at the second junction, as illustrated in Figure 14c. Rhodamine B-doped fluids are perfused in the outer and outer three pipes, facilitating the smooth movement of the four fluids through the final junction while maintaining clear boundaries between each fluid, as shown in Figure 14d.

### 3.5. Preparation and Characterization of Fibers

Microfluidics technology can integrate complex analysis and detection processes in a small volume and can realize sampling, mixing, separation, enrichment, and other operations. It can provide a fast, accurate, and automated platform at a low cost, which can be used in wearable devices [33], biomarker diagnostics [34,35,36,37], and the preparation of gel fibers.

**Figure 15:**
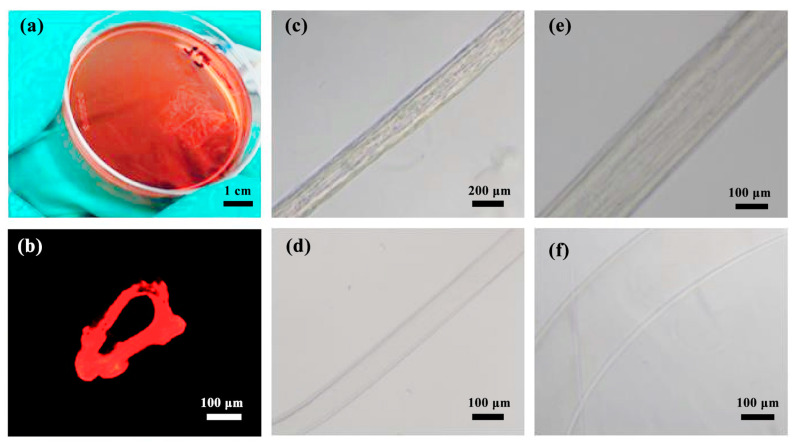
Preparation of hollow fiber: (**a**) fiber collection; (**b**) Cross-section fluorescence diagram of gel fiber; (**c**) fiber diagram generated when the intermediate fluid is PVA solution (40×); (**d**) A fiber diagram is generated when the intermediate fluid is CaCl_2_ solution (40×); (**e**) A fiber diagram is generated when the intermediate fluid is PVA solution (100×); (**f**) A fiber diagram is generated when the intermediate fluid is CaCl_2_ solution (100×).

In order to characterize the laminar flow phenomenon, this paper uses the injection method shown in Figure 2 to inject the fluid into the microfluidics chip, maintain the flow rate of both the inner and outer layers at 1.0 mL/h, and collect the generated fibers with a clean Petri dish. The pigment diffuses in a 2 wt% CaCl_2_ solution and turns red (Figure 15a,b). When the intermediate fluid is 10 wt% PVA solution, there is an obvious filamentous structure in the middle part of the fiber (Figure 15c,e) because the PVA solution has a high viscosity and a slow flow, and it is difficult to flow out of the collected fiber smoothly, which affects the cross-linking of sodium alginate and calcium ions.

When the intermediate fluid is replaced with a 0.1 wt% CaCl_2_ solution, the low-concentration calcium ion can be pre-crosslinked with sodium alginate, and the pipeline will not be blocked due to the direct crosslink between calcium ion and sodium alginate due to the excessive concentration of calcium ion. A clear hollow structure with a thin fiber wall can be obtained (Figure 15d,f).

In order to better observe the cross section of the fiber, the gel fiber containing fluorescent dyes is frozen and sliced with a thickness of 20 μm. The hollow structure of the fiber cross-section can be seen through a fluorescence microscope, and the circular shape of the cross-section is slightly deformed, possibly caused by compression during freeze-cutting (Figure 15b).

## 4. Summary

This paper presents the design of a microfluidic chip that eliminates the need for hydrophilic modification of microchannels and offers a convenient operation process. A series of comparative experiments were conducted to investigate the effects of different fluid compositions and flow rates. The results indicate that the utilization of sodium alginate as the outer solution, CaCl_2_ as the inner solution, and a flow rate of 1 mL/h for both layers enables the preparation of hollow structural fibers with superior morphology. Furthermore, this configuration ensures the most stable fluid movement and the occurrence of distinct laminar flow phenomena. The findings of this study serve as a valuable reference for exploring the influence of flow rate on the laminar flow phenomenon in microfluidic chips.

## Figures and Tables

**Table 1:** Experiment 1: Flow rate data.

	Group	1	2	3	4	5
Flow Rate (mL/h)						
Intermediate velocity		0.1	0.2	0.3	0.4	0.5
Outer layer velocity		0.2	0.4	0.6	0.8	1.0

**Table 2:** Experiment 2: Flow rate data.

	Group	1	2	3	4	5	6	7	8	9	10
Flow Rate (mL/h)											
Intermediate velocity		0.1	0.2	0.3	0.4	0.5	0.6	0.7	0.8	0.9	1.0
Outer layer velocity		0.1	0.2	0.3	0.4	0.5	0.6	0.7	0.8	0.9	1.0

**Table 3:** Experiment 3: Flow rate data.

	Group	1	2	3	4
Flow Rate (mL/h)					
Intermediate velocity		1.0	1.0	1.0	1.0
Outer layer velocity		0.4	0.6	0.8	1.0

## Data Availability

Data sharing is not applicable to this article.
